# A novel method for evaluating the dynamic biocompatibility of degradable biomaterials based on real-time cell analysis

**DOI:** 10.1093/rb/rbaa017

**Published:** 2020-05-01

**Authors:** Xiaoxiao Gai, Chenghu Liu, Guowei Wang, Yang Qin, Chunguang Fan, Jia Liu, Yanping Shi

**Affiliations:** r1 Department of Biological Evaluation, Shandong Quality Inspection Center for Medical Devices, NO.15166 Century Avenue, H-T Industrial Development Zone, Jinan 250101, China; r2 Shandong Key Laboratory of Biological Evaluation for Medical Devices, NO.15166 Century Avenue, H-T Industrial Development Zone, Jinan 250101, China; r3 Department of Biological Evaluation, NMPA Key Laboratory for Safety Evaluation of Biomaterials and Medical Devices, NO.15166 Century Avenue, H-T Industrial Development Zone, Jinan 250101, China

**Keywords:** degradable biomaterials, biocompatibility, real-time cell analysis, magnesium-based biomaterials

## Abstract

Degradable biomaterials have emerged as a promising type of medical materials because of their unique advantages of biocompatibility, biodegradability and biosafety. Owing to their bioabsorbable and biocompatible properties, magnesium-based biomaterials are considered as ideal degradable medical implants. However, the rapid corrosion of magnesium-based materials not only limits their clinical application but also necessitates a more specific biological evaluation system and biosafety standard. In this study, extracts of pure Mg and its calcium alloy were prepared using different media based on ISO 10993:12; the Mg^2+^ concentration and osmolality of each extract were measured. The biocompatibility was investigated using the MTT assay and xCELLigence real-time cell analysis (RTCA). Cytotoxicity tests were conducted with L929, MG-63 and human umbilical vein endothelial cell lines. The results of the RTCA highly matched with those of the MTT assay and revealed the different dynamic modes of the cytotoxic process, which are related to the differences in the tested cell lines, Mg-based materials and dilution rates of extracts. This study provides an insight on the biocompatibility of biodegradable materials from the perspective of cytotoxic dynamics and suggests the applicability of RTCA for the cytotoxic evaluation of degradable biomaterials.

## Introduction

The medical application of metallic biomaterials has a long history, and these biomaterials are still widely used in modern orthopedic surgery because of their high impact strength, ductility and corrosion resistance [1, 2]. At present, stainless steels, cobalt–chromium alloys and titanium alloys are commonly used as metallic implant materials [3, 4]. However, various studies have reported their disadvantages such as stress shielding caused by the density difference between the metal implants and the newly generated bone tissues, as well as the release of toxic ions (e.g. from titanium particles) through corrosion or wear [5–7]. Among these disadvantages, the most undesirable one is non-degradability, which makes it necessary to perform a secondary surgery after bone fixation, thus increasing the patient suffering and the possibility of postoperative infection [8, 9]. Therefore, degradable biomaterials have drawn increasing interest and intensive studies have been conducted in recent years owing to their unique advantage of avoiding secondary surgery for implant removal [10, 11].

Medical implants based on degradable biomaterials can provide fixation and reinforcement to human bones and tissues for the required period and subsequently degrade and dissolve in the human body. Several studies have focused on developing biodegradable implants based on different materials such as high molecular polymers, ceramics and degradable metals [10, 12–14]. Among these materials, magnesium-based biomaterials are promising owing to their bioabsorbable properties, low density and high strength, as well as their remarkable bioactivity, which induces new bone formation and stimulates angiogenesis—the two relevant processes crucial for the healing of bone fractures [6, 15, 16]. Apart from the abovementioned advantages, magnesium is an essential trace nutrient element and the fourth most abundant cation in the human body; therefore, biomaterials based on magnesium have an inherent advantage of biocompatibility [17]. In spite of their superiority over other orthopedic materials, the fast degradation of Mg-based medical implants accompanied by the deterioration of their mechanical properties caused due to rapid corrosion has limited their clinical applications [18]. The microstructure and the degradation rate of pure magnesium can be modified via alloying. Moreover, suitable chemical elements can be used to enhance the mechanical and physical properties of the microstructure [19, 20]. Mg-based alloys with corrosion resistance possess good mechanical compatibility and yield higher initial stability and initial support, when compared with pure magnesium [21]. Commonly used alloying elements include calcium, aluminum, zinc, some metal salts and some rare-earth elements. It has been reported that the Mg-Ca alloy and bioactive glass-Mg composites exhibit improved biomechanical properties, increased biodegradability and low corrosion and can also stimulate the differentiation and proliferation of osteoblastic cell lines [21–23]. Further investigations on novel injectable Mg/Ca sulfate hemihydrate (Mg/CSH) composites have indicated enhanced mechanical properties and high degrees of cell attachment, proliferation and osteogenic differentiation, when compared with those of CSH composites [24]. Therefore, Mg alloys are regarded as suitable materials for a new generation of medical implants with promising prospects of clinical applications. However, due to the risk attributed to the high degradability of medical implants based on Mg and its alloys, a systematic evaluation of the biosafety and efficacy and physicochemical characteristics must be performed by government regulatory and testing bodies employing a standard protocol, prior to product registration and market access approval.

Biological compatibility is the fundamental indicator for the biosafety evaluation of medical implants. Currently, a majority of the techniques for evaluating the cytotoxicity of degradable implants apply the indirect methods of the ISO 10993 series of standards [25, 26]. According to these standards, the MTT assay is the suggested cytotoxicity test for the biocompatibility evaluation of biodegradable materials. However, these test standards were originally developed for non-degradable materials. Unlike inert metals, Mg-based metals can instantly react with water and release significant amounts of Mg^2+^, which has high affinity with hydroxyl, accompanied by an increase in free hydrogen that increases the pH value and osmolality of the surrounding medium. Therefore, it is difficult to accurately evaluate the intrinsic cytotoxicity of magnesium alloys [27]. Previous studies have revealed that preparing the extracts of biodegradable implants could significantly influence the outcomes of *in vitro* cytotoxicity tests [9, 27]. To mimic the *in vivo* environment, several improved extract preparation methods were applied based on Parts 5 and 12 of the ISO 10993 standard, including cell culture mediums supplemented with serum and extract dilution [9, 27, 28]. Although the cytotoxic information obtained from those methods has high reliability, it only represents the cell viability and proliferation at a specific time after treatment with various extracts of the implant. Considering the significant difference in corrosion rate and degradation dynamics among various Mg-based biomaterials [29, 30], the dynamic variation information of cell properties of such implants was indispensable to further understand their biocompatibility.

Flow cytometry, spectrometry and microscopy analyses have been widely used for cytotoxicity evaluation to determine the cellular condition of the specific treatment or the environment. However, such assays could only reflect the physiological and morphological traits of cells at the end of treatment or a certain time point; they are incapable of providing an accurate depiction of the dynamic cellular process during the analysis [31–34]. The xCELLigence real-time cell analysis (RTCA) system is an impedance-based microelectronic cell sensor technology designed for a label-free and real-time monitoring of cell properties. It is noninvasive and has a high efficiency [35]. Moreover, it has the ability to detect cellular adherence, proliferation, migration and cytotoxicity [36, 37] and is mainly applied in medical research where human cancer cells and cardiomyocyte are the objects [38–40]. Therefore, the RTCA is capable of performing dynamic cellular tests with ease and reproducible accuracy compared to conventional endpoint cellular assays. This unique advantage of the RTCA system indicates its significant potential in biocompatibility tests of medical devices. To our knowledge, the application of RTCA in the cytotoxic study of degradable biomaterials has rarely been reported.

This study was conducted based on ISO 10993-5:2009: biological evaluation of medical devices—part 5: tests for *in vitro* cytotoxicity. Extracts of pure Mg and its Ca alloy were prepared with different cell media according to ISO 10993:12, and the Mg^2+^ concentration and osmolality of the extracts were measured. Cytotoxicity tests were conducted with human osteosarcoma cell line MG-63, human umbilical vein endothelial (HUVE) cell line and mouse fibroblast cell line L929, based on ISO 10993:5. In addition, the cytotoxicity dynamics of Mg and its alloy with tested cells were determined using the xCELLigence RTCA system. The main purpose of the present study was to improve the accuracy and validity of existing cytotoxic evaluation methods for Mg-based biodegradable implants with a focus on the dynamic characteristics of cytotoxicity.

Our results suggested the applicability of the RTCA system for the cytotoxic evaluation of biodegradable materials. It was also revealed that the effects of the material extracts preparation as well as the dynamic characteristics of cytotoxicity should be taken into consideration to achieve an accurate and valid cytotoxicity evaluation of biodegradable materials *in vitro*.

## Materials and methods

### Specimen preparation

Pure magnesium (99.99%) was bought from China New Metal Materials Technology Co. Ltd., and magnesium alloy with 0.067 wt.% calcium (MgCa 0.067) was bought from MBH Analytical Ltd.

### Extract preparation

The pure magnesium (99.99%) and MgCa 0.067 were sterilized for 15 min at 115°C. Subsequently, test specimens were incubated with three cellular culture media, respectively: minimal essential medium (MEM), Dulbecco’s modified Eagle’s medium (DMEM) and CM15-1 (90% F-12 K + 10% FBS + 0.1 mg/ml heparin + 0.03–0.05 mg/ml ECGS). All the media were supplemented with 10% fetal bovine serum (Sijiqing, China). The incubation was conducted in a cell incubator for 72 h at conditions of 5% CO_2_, 20% O_2_, 95% humidity and 37°C. Here, an extract was prepared with the ratio of the specimen weight to the extraction medium as 0.2 g/ml according to EN ISO 10993-12:2009 (sample preparation and reference materials), and the extract was called 1×. Less concentrated extracts were prepared using the same procedure with different dilution times; the extract diluted two times by the extraction medium was called 2×. Other extracts (4×, 8×, 16× and 32×) were prepared with same procedure, and all extracts were filtered.

### Osmolality and magnesium concentration measurement

The osmolality of the extracts was measured using Löser OM815 cryoscopic osmometer (Löser, Germany). The analyzed extract volume was 100 µL. The PE Avio 200 inductively coupled plasma optical emission spectrometer (ICP-OES, PE, USA) was used for a quantitative determination of the magnesium concentration in the different extracts. The operation parameters of the ICP-OES were set as follows: working mode: radial plasma; gas flow = 10 ml/min; auxiliary gas flow = 0.2 l/min; nebulizer gas flow = 0.7 l/min; pump flow = 1.50 ml/min; ICP RF power = 1350 W and spectral line = Mg—285.213 nm. The gradient concentration of the reference standard Mg^2+^ solution was prepared as 1, 2, 5, 8, 10 and 20 mg/l based on 100 µg/ml of magnesium storage solution. And pH value of extracts was determined by an acidometer (Delta 320, Mettler Toledo, Switzerland). The analyses of osmolality and Mg^2+^ concentration strictly followed the standard procedure of Pharmacopoeia of the People’s Republic of China 2015 (ChP 2015).

### Cell culture

#### MG-63 cell line

The human osteosarcoma cell line MG-63 was obtained from The Cell Bank of Type Culture Collection of Chinese Academy of Sciences. The cells were cultured in DMEM, supplemented with 10% fetal bovine serum (Sijiqing, China) in a cell incubator under 5% CO_2_ and 37°C. After thawing from stock, the cells were passaged two to three times at 80% confluency before subjecting them to cytotoxic tests. To maintain the sensitivity, the cells were only sub-cultured for up to 20 passages.

#### L929 cell line

The L929 mouse fibroblast cell is a mammalian cell line, i.e. widely used for the *in vitro* cytotoxicity evaluation of biomaterials and medical devices. In this study, L929 cells were obtained from Conservation Genetics CAS Kunming Cell Bank. The cells were cultured in MEM supplemented with 10% fetal bovine serum (Sijiqing, China) in a cell incubator at 5% CO_2_ and 37°C. The test used a passage of 48–72-h vigorous growth of cells.

#### HUVE cell line

The HUVE cells were obtained from BeNa Culture Collection, China. The cells were cultured in CM15-1 (90% F-12 K + 0.1 mg/ml heparin + 0.03–0.05 mg/ml ECGS) and supplemented with 10% fetal bovine serum (Sijiqing, China) in a cell incubator at 5% CO_2_ and 37°C. The cells were passaged at 80% confluency, and cells after the fifth passage were used in the experiment.

### Cytotoxicity evaluation

#### MTT assay

L929, MG-63 and HUVE cell suspensions were prepared with the cell culture medium and the density was adjusted to 1 × 10^5^ cells/ml. A cell suspension of 100 μl was added in a 96-well plate and the plate was placed in a cell incubator (5% CO_2_ and 37°C) for 24 h to form a sub-confluent cell monolayer. Each plate was examined under a microscope to ensure that the cell growth was relatively even across the microtiter plate. Subsequently, the culture medium was replaced with extracts and incubated under the cell culture conditions. After 48 h of incubation, the morphological traits of the cells in each well were examined with an inverted microscope (Olympus, Japan) and recorded. Afterward, the culture medium was carefully removed from the plates. Thereafter, 50 µL of the MTT solution was added to each test well and the plates were further incubated for 2 h at 37°C. Subsequently, the MTT solution was decanted and 100 µL of isopropanol was added in each well. The plates were swayed and the absorbance was measured with a microplate reader at a wavelength of 570 nm (the reference wavelength was 650 nm). The cell viability (%) was calculated by the following formula:

$$\text{Viab}.\%=\frac{100\times {\text{OD}}_{570}{}_{e}}{{\text{OD}}_{570b}}$$

where OD_e_ refers to the absorption of the sample, and OD_b_ refers to the absorption of the vehicle control.

### Real-time cell analyzer test

A culture medium of 150 μl was added to the E-plate of the RTCA system and the cell index (CI) background value of the medium was measured. L929, MG-63 and HUVE cell suspensions were prepared with the cell culture medium and the density was adjusted to 1 × 10^5^ cells/ml. Subsequently, the culture medium was decanted, and a 300-μl cell suspension was added to the E-plate before the software schedule was initiated for 30 min. The E-plate was put into the RTCA cradle (the RTCA cradle was placed in the incubator in advance) to conduct real-time and dynamic cell proliferation detection. Impedance signals were recorded every 20 min for the first 12 scans (4 h) and every 15 min for the second 80 scans (20 h). After 24 h, the old culture medium was replaced with extracts and the experiment was run for another 48 h. The impedance signals were recorded every 1 h until the end of the experiment. The CI value was given by the RTCA software package based on the impedance signal.

### Statistical analysis

For each assay, triplicate experiments were performed, and the significance of group differences was examined using Student’s two-tailed *t*-test (Microsoft Excel software).

## Results and discussion

### Osmolality and magnesium concentration of extracts with different ratios of magnesium-based samples and media

**Figure 1:**
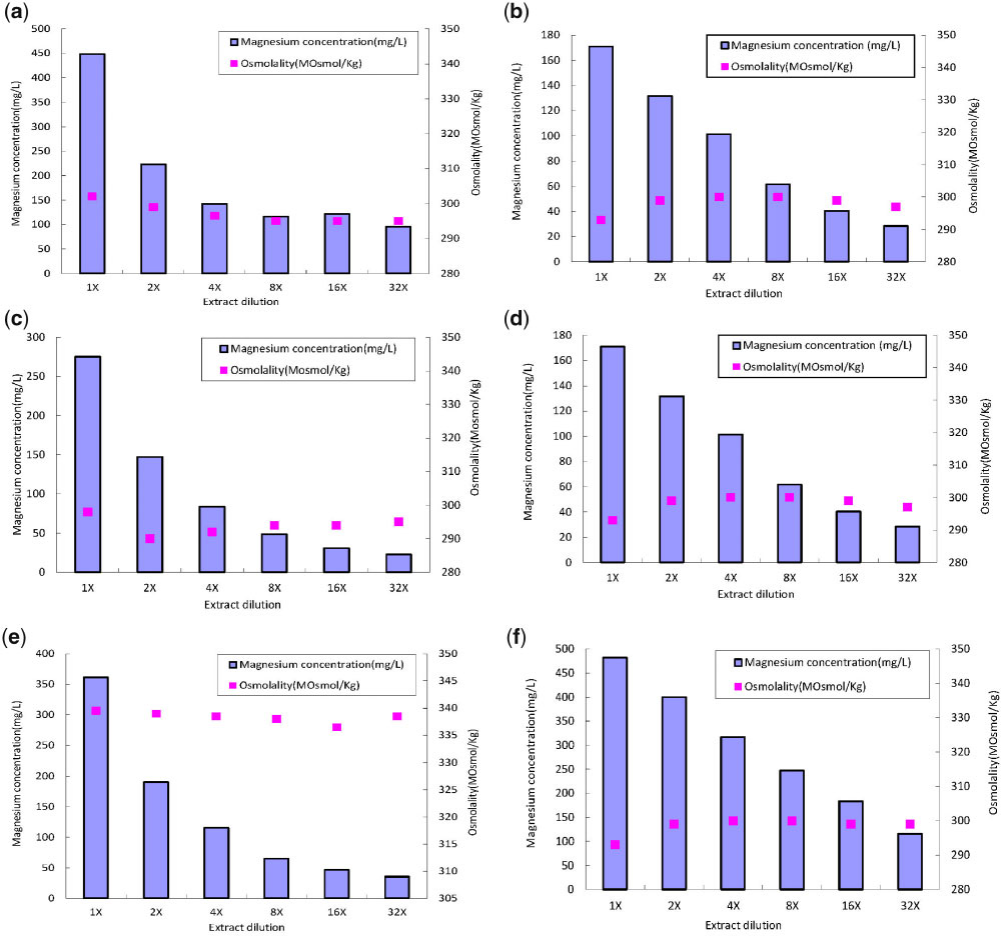
Mg^2+^ concentration and osmolality of three media with different dilution level of extracts prepared from magnesium alloys and pure magnesium. CM15-1 media with pure magnesium (**a**) and magnesium alloys (**b**); MEM media with pure magnesium (**c**) and magnesium alloys (**d**) and DMEM media with pure magnesium (**e**) and magnesium alloys (**f**).

The osmolality and magnesium concentration of the extracts were analyzed using the cryoscopic osmometer and ICP-OES, respectively. As shown in Fig. 1, the content difference of magnesium ions (Mg^2+^) in the three cellular media is apparent as the dilution ratio changed. The magnesium concentrations of the pure magnesium extracts (1×) of the three media are 4.2, 4.7 and 7.1 times higher than that of the heavily diluted extracts (32×), respectively, whereas the osmolality shows no significant difference among different dilution ratios. The Mg^2+^ concentrations of extracts prepared from pure magnesium and magnesium–calcium alloy also exhibit a noticeable difference. The osmolality of the solution system is dependent on its osmotic potential, which is determined by the total particle or ion quantity of the system. According to the laws of thermodynamics, the potential energy state of a system with more particles is low due to the effect of the free energy offset caused by molecular collision. In the present study, the magnesium-based samples were extracted by different cellular culture media, which contain a lot of organic and inorganic molecules to sustain the homeostasis conditions of the *in vitro* culture environment. These molecules play crucial roles in the cell cycle [27]. Under such circumstances, given the high molecular quantity background of the extract medium, the impact of the increase in magnesium ions caused by magnesium corrosion on the osmolality of the extract system was offset mostly by the culture medium.

**Figure 2:**
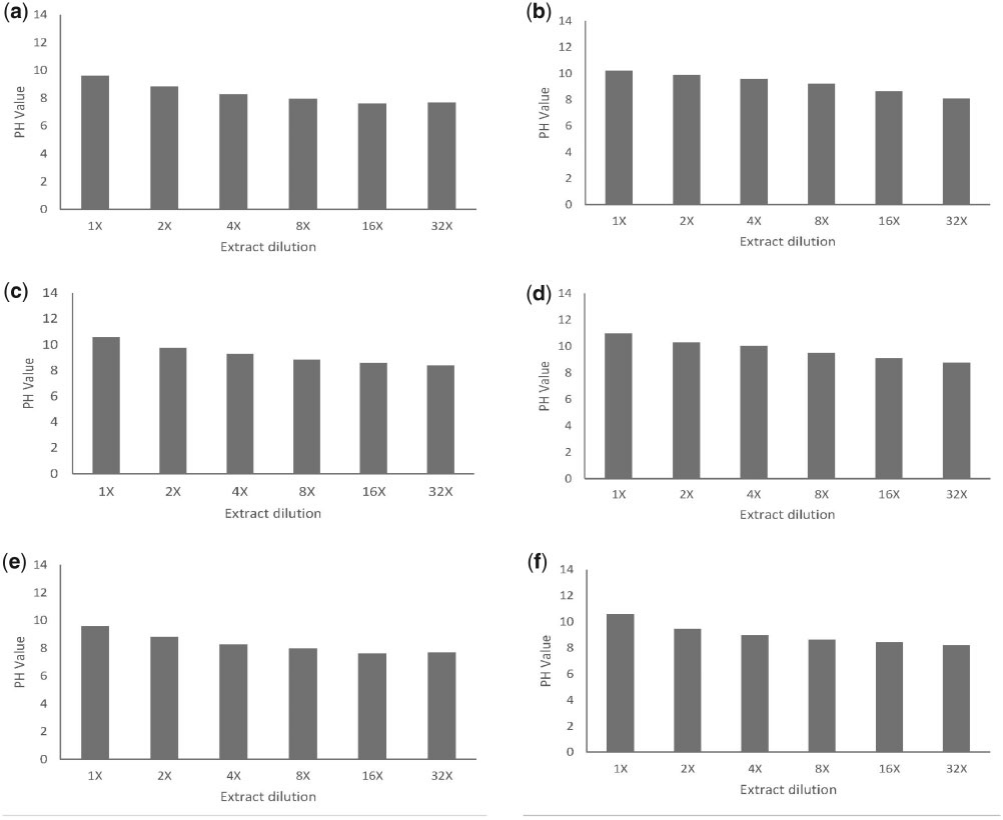
pH value of cell culture media with different dilution level of extracts prepared from pure magnesium and magnesium alloys. CM15-1 media with pure magnesium (**a**) and magnesium alloys (**b**); MEM media with pure magnesium (**c**) and magnesium alloys (**d**); and DMEM media with pure magnesium (**e**) and magnesium alloys (**f**)

On the other hand, the results of pH analysis shown in Fig. 2 indicate that the relative stable pH values of the heavily diluted extracts (4×, 8×, 16× and 32×) are consistent with the osmolality results. It is known that magnesium undergoes oxidation reactions in aqueous solutions, generating hydrogen and hydroxyl due to the electron transfer from Mg to H_2_O and resulting in an increase in the pH value [8, 41]. A previous study reported the impact of pH change on a Mg-based biodegradable material; thus, pH buffering should be considered to achieve an accurate cytotoxic evaluation of biodegradable materials [27]. In the present study, the tested samples were prepared with a complete cell medium; therefore, the consistency of pH value was maintained by the acid–base buffer pair of the medium in the different diluted extracts with dissimilar Mg concentration. Hence, the results of the osmolality and pH value suggested that the intrinsic cytotoxicity of the magnesium alloy could be the main factor causing the cellular stress rather than the pH and osmolality change caused by corrosion in the heavily diluted extracts.

### Cytotoxicity of extracts of Mg and Mg-based alloy

**Figure 3:**
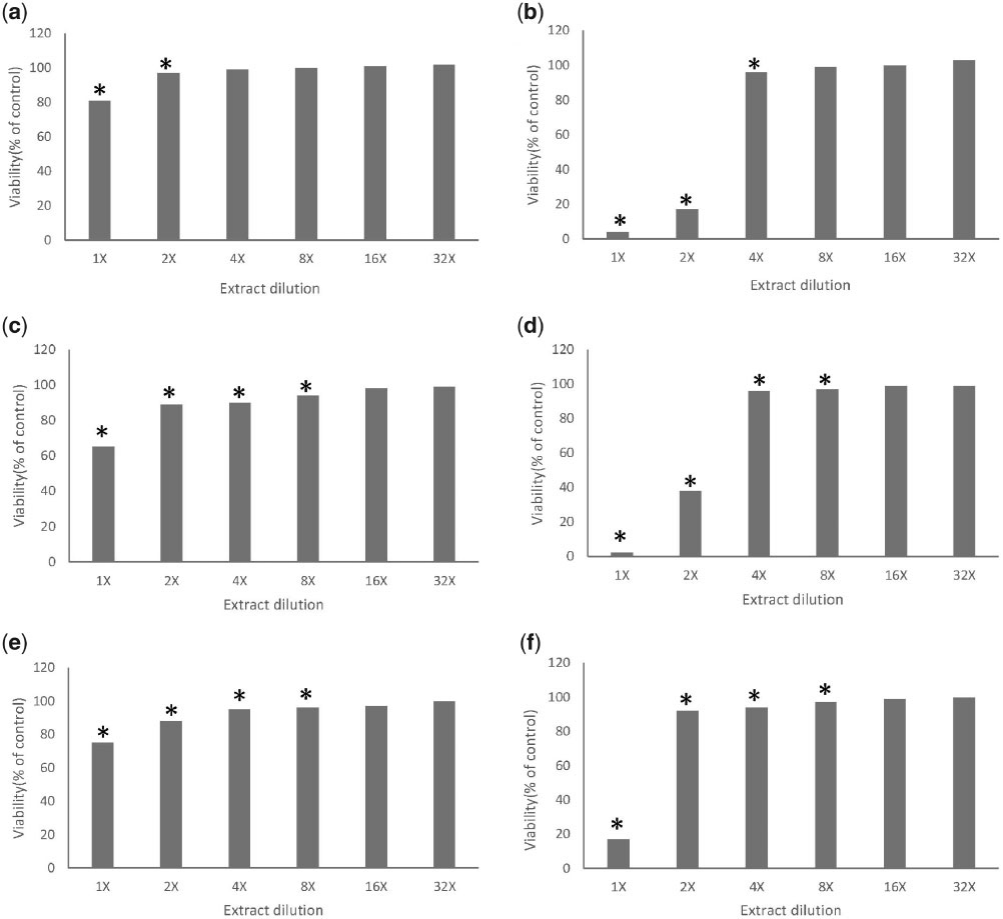
The cell viability of three cell lines with different dilution level of extracts of pure magnesium and magnesium alloys. HUVE cell line with pure magnesium (**a**) and magnesium alloys (**b**); L929 cell line with pure magnesium (**c**) and magnesium alloys (**d**); and MG63 cell line with pure magnesium (**e**) and magnesium alloys (**f**) (cell viability was presented as percentage of blank control; *P <* *0.01, when compared with the control)

The results of the MTT assay are illustrated in Fig. 3. The magnesium–calcium alloy extract exhibits a significantly stronger inhibition effect on all the tested cell lines compared to the extract of pure magnesium in high concentration, and a relatively similar inhibition pattern can be observed in the other diluted extracts. The inhibition rates of the 1× magnesium–calcium alloy extracts to the three cell lines are all above 80%, whereas the inhibition rates of the 1× pure magnesium extracts are all less than 40%. The MTT assay is a common method employed in most of the cytotoxic evaluations of biomaterials that reflect the dose–response relationship of the tested sample according to the ISO standard 10993-5 [25, 26]. However, the MTT is a typical endpoint assay determined by the activities of NAD(P)H-dependent dehydrogenase enzymes-succinate dehydrogenase at a certain time point, and it is incapable of reflecting the dynamic characteristics of the cytotoxic process [42–44]. For instance, the MTT results of this study indicated the sensitivity difference of the three cell lines to the same extract and the different cytotoxic behaviors of different extracts to the same cell line. However, to better understand the cytotoxic mechanism, the dynamic information of these cytotoxic processes is necessary.

**Figure 4:**
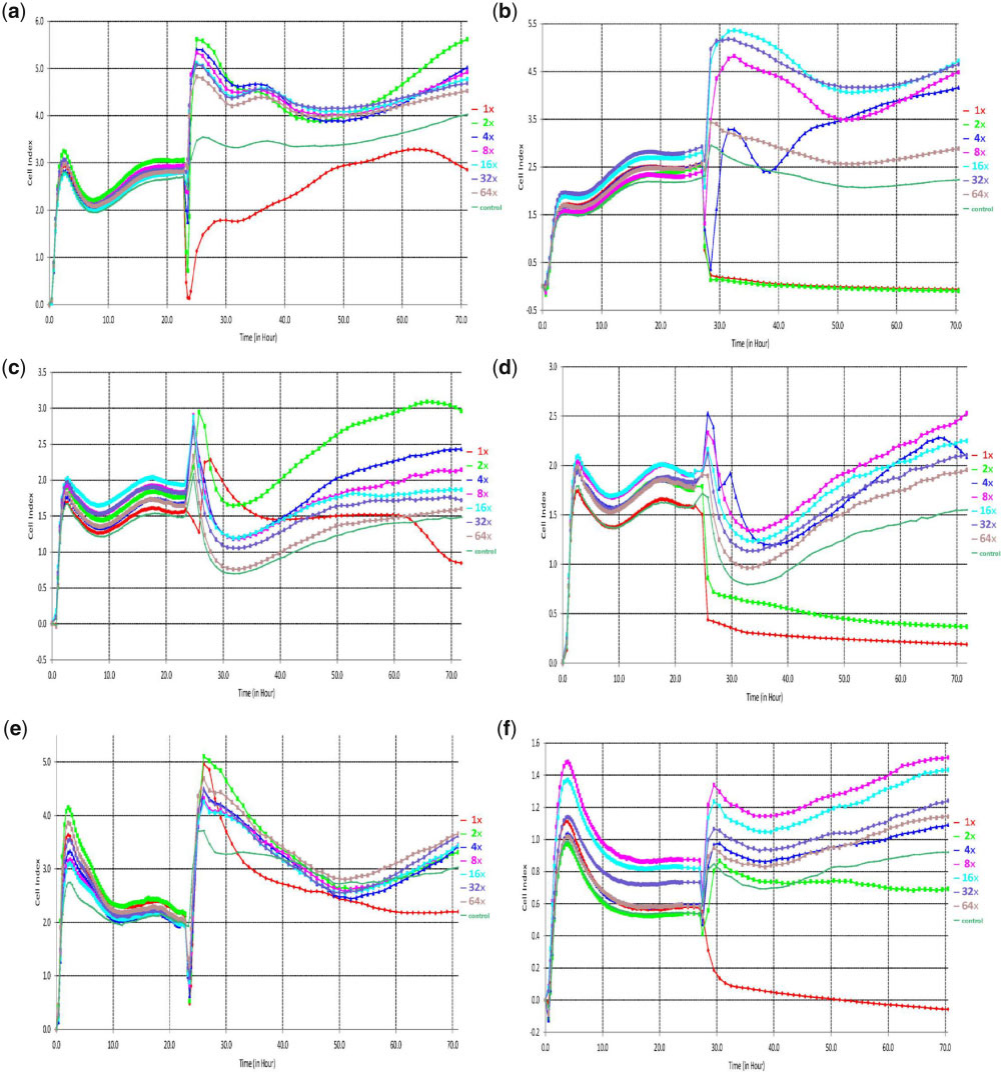
The cell index of three cell lines with different dilution level of extracts prepared from pure magnesium and magnesium alloys. HUVE cell line with pure magnesium (**a**) and magnesium alloys (**b**); L929 cell line with pure magnesium (**c**) and magnesium alloys (**d**); and MG63 cell line with pure magnesium (**e**) and magnesium alloys (**f**)

The real-time analysis of the proliferation of the L929, MG-63 and HUVE cell lines was conducted using the xCELLigence RTCA system. The results of the RTCA are shown in Fig. 4, after cells were seeded into E-plate, the CI curve of 0–24 h represents the process of cell adherence and proliferation and the fluctuation of the CI values was observed in three cell lines cultured with extracts of Mg and Mg-based alloy in a gradient dilution rate. The obvious differences in the CI variation trends from 48 h indicate the proliferation differences among the three cell lines with time. According to the time sequence of the minimum CI value and average CI value, the high-concentration Mg–calcium alloy extracts (1× and 2×) show the highest cytotoxicity to the tested cell lines and the most significant differences in cell proliferation behavior with time. In contrast, the pure magnesium extracts with different dilution rates show a relatively similar cell proliferation behavior with time. The CI value represents the changing impedance of the E-plate electrodes, which correlates with the quantity and quality of cell interactions and adherent properties between the cell and electrodes [45]; therefore, the variation trend of the CI value can provide a more comprehensive insight on the biocompatibility of the biodegradable material during the entire experimental period [46, 47]. The value of CI can be used to depicted several cellular processes, e.g. cell viability, cell proliferation, adhesion degree, thus the fluctuation of CI value can provide the information of cellular physiological states including cell growth, morphological changes, the extent of cell death and cell adhesion which contribute to further understanding of cytotoxic mechanism of biomaterials [48, 49]. Typically, in the previous study, some researchers have also reported that the specificity and sensitivity of RTCA are 99.6 and 87.5%, respectively [51].

In this study, combining the results of the RTCA, MTT assay and pH value, it was found that the intrinsic cytotoxicity of the Mg-based alloy metal ion is the main factor causing the cellular stress rather than the hydroxyl and Mg ion. In addition, same as previous reports [51, 52], the overall cytotoxic results of the RTCA match those of the MTT assay. Therefore, the consistent result of MTT assay and RTCA suggests the applicability of the RTCA in the cytotoxic evaluation of degradable biomaterials. Furthermore, with the future study of wider range of materials and improvement in methodology, a novel method could be established based on RTCA technology and provides a complementary method for the corresponding ISO standard of biocompatibility evaluation of medical biomaterials.

## Conclusion

The biocompatibility of degradable biomaterials based on magnesium and its alloy was investigated using two cytotoxic evaluation methods: the conventional MTT assay and RTCA. The main purpose of this study was to improve the accuracy and validity of existing methods to evaluate the biocompatibility of Mg-based degradable biomaterials with a focus on cytotoxic dynamics. The result of the MTT assay revealed the cytotoxic difference between pure magnesium and its alloy. The inhibition rates of high-concentration Mg alloy extracts exceeded 80% in all cell lines, whereas those of pure magnesium extracts with the same concentration were all less than 40%. The results of the RTCA revealed the different dynamic modes of the cytotoxic process, which are related to the differences in the tested cell lines, Mg-based materials and dilution rates of extracts. In addition, the overall cytotoxic results of the RTCA were consistent with those of the MTT assay and suggests the application prospect of RTCA in biocompatibility evaluation of medical biomaterials.

This study revealed the biocompatibility of Mg-based biodegradable materials from the perspective of cytotoxic dynamics. Furthermore, it demonstrated that the RTCA is suitable for the cytotoxic evaluation of degradable biomaterials, and future study will be conducted in wider range of biomaterials to establish and improve the methodology of RTCA, and hence provide scientific data to the further emendation of ISO standard for biocompatibility evaluation of medical biomaterials.

## Funding

This work was supported by the National Key Research and Development Project of China (NO. 2016YFC1103205).

*Conflict of interest statement.* None declared.
